# Early-season and refined mapping of winter wheat based on phenology algorithms - a case of Shandong, China

**DOI:** 10.3389/fpls.2023.1016890

**Published:** 2023-07-24

**Authors:** Xiuyu Liu, Xuehua Li, Lixin Gao, Jinshui Zhang, Dapeng Qin, Kun Wang, Zhenhai Li

**Affiliations:** ^1^ State Key Laboratory of Remote Sensing Science, Beijing Normal University, Beijing, China; ^2^ Institute of Remote Sensing Science and Engineering, Faculty of Geographical Science, Beijing Normal University, Beijing, China; ^3^ College of Geodesy and Geomatics, Shandong University of Science and Technology, Qingdao, China; ^4^ Roquette Management (Shanghai) Com. Ltd, Shanghai, China; ^5^ Key Laboratory of Digital Earth Science, Aerospace Information Research Institute, Chinese Academy of Sciences, Beijing, China

**Keywords:** winter wheat, early mapping, phenological information, contemporaneous crops, Sentinel-2

## Abstract

Winter wheat is one of the major food crops in China, and timely and effective early-season identification of winter wheat is crucial for crop yield estimation and food security. However, traditional winter wheat mapping is based on post-season identification, which has a lag and relies heavily on sample data. Early-season identification of winter wheat faces the main difficulties of weak remote sensing response of the vegetation signal at the early growth stage, difficulty of acquiring sample data on winter wheat in the current season in real time, interference of crops in the same period, and limited image resolution. In this study, an early-season refined mapping method with winter wheat phenology information as priori knowledge is developed based on the Google Earth Engine cloud platform by using Sentinel-2 time series data as the main data source; these data are automated and highly interpretable. The normalized differential phenology index (NDPI) is adopted to enhance the weak vegetation signal at the early growth stage of winter wheat, and two winter wheat phenology feature enhancement indices based on NDPI, namely, wheat phenology differential index (WPDI) and normalized differential wheat phenology index (NDWPI) are developed. To address the issue of “ different objects with the same spectra characteristics” between winter wheat and garlic, a plastic mulched index (PMI) is established through quantitative spectral analysis based on the differences in early planting patterns between winter wheat and garlic. The identification accuracy of the method is 82.64% and 88.76% in the early overwintering and regreening periods, respectively, These results were consistent with official statistics (R2 = 0.96 and 0.98, respectively). Generalization analysis demonstrated the spatiotemporal transferability of the method across different years and regions. In conclusion, the proposed methodology can obtain highly precise spatial distribution and planting area information of winter wheat 4_6 months before harvest. It provides theoretical and methodological guidance for early crop identification and has good scientific research and application value.

## 1 Introduction

Food security is critical for the worldwide community, national economic development, social harmony, and people’s daily lives. Food security is eliciting increasing attention as a result of urbanization, global climate change, and the loss of farmland due to deterioration (Liu et al., 2014; Song et al., 2018). With the rapid growth of Earth observation data in the past decades, remote sensing has been widely recognized in informative agricultural applications because of its advantages of broad spatial coverage, high revisit frequency, low cost and simple accessibility (Griffiths et al., 2019; Jin et al., 2019). Remote sensing technology can perform timely and accurate mapping of crop types with high accuracy, and it has been proven to be one of the most effective ways to determine the spatial distribution of crop cultivation (Pan et al., 2012; Griffiths et al., 2019). Crop identification and crop acreage estimation in early or in-season can help in crop cultivation management, food security scenario analysis and related policy formulation and also have important applications in crop yield forecasting, agricultural insurance, agricultural subsidies and agricultural restructuring.

Currently, the identification of crop types usually requires the use of image data on their entire growth period, so the results of crop distribution maps are often obtained after the season or in the following year, with a certain delay. For example, the United States Department of Agriculture Cropland Data Layer (CDL) Dataset is published about five months after the end of the crop harvest, and the Agriculture and Agri-Food Canada Annual Crop Inventory (ACI) is usually published about eight months after harvest (Fisette et al., 2014; Kussul et al., 2018). Although CDL and ACI datasets have high accuracy, they both have time delay. Therefore, the importance of in-season or early-season crop type mapping based on remote sensing has become a valuable research topic.

The amount of information available for early crop remote sensing identification is smaller than that for post-season identification. The first manifestation is the reduction in remote sensing data, which are limited to the early crop growth time period. At the early stage of crop growth, the characteristic response on the remote sensing image of crop growth is not significant due to the effect of mixed image units caused by vegetation and soil; therefore, fully exploiting the phenological and spectral information in the early stage of crop growth can contribute to highly accurate early crop mapping. Dong et al. (2016) developed a pixel-level rice mapping method based on the Google Earth Engine (GEE) platform by using the water signal characteristics at the rice transplanting stage, and accomplished rice mapping in Northeast China by using the enhanced vegetation index (EVI) and the land surface water index (LSWI). Basing on the fact that corn is seeded earlier than soybean in the United States, Sakamoto et al. (2014) accomplished mapping and yield calculation for both crops. The second manifestation is that early identification studies have little opportunity to obtain sample dates in the current year (Johnson and Mueller, 2021). A common strategy is to use a migration learning approach that utilizes sample data from previous years to train the model and apply it to the current year. Cai et al. (2018) completed early-season mapping of maize and soybean by using a transfer learning algorithm based on Landsat time series data. Zhang et al. (2019) adopted artificial neural networks to predict the spatial distribution of future crop plantings before the start of the growing season on the basis of historical information and effectively completed pre-season crop mapping and crop yields estimation for a normal year.

In addition to the reduction in available remote sensing information, the spatial resolution of remote sensing images is another factor that affects identification accuracy. In previous studies, MODIS data were the main data source for crop mapping in large regions. Hao et al. (2015) employed MODIS time series data to investigate the effect of time length on crop mapping by using Kansas, USA, as the study area and achieved high crop identification accuracy after five months of the sowing period. However, most of China’s cultivated land is fragmented, and villages, towns, and wheat are interspersed. The average planting area per household is only 1.37 hectares, which account for 5% of the MODIS image size, and the planting structure is complex. Moreover, the mixed image phenomenon at the boundary of the land is serious, so accurately identifying the winter wheat planting area is difficult (Zhang, 2008; Qiu et al., 2017). Tian et al. used multi-source remote sensing data based on a phenological algorithm to map the crop distribution and subsequently compared and analyzed the difference in the accuracy of identifying winter wheat by using Landsat-7, Landsat-8, and Sentinel-2 remote sensing data and MODIS data (including 250 and 500 m) under the same method. The results showed that image spatial resolution has a considerable influence on the remote sensing recognition results, and the mapping accuracy increases with the increase in spatial resolution. Moreover, MODIS data cannot accurately identify village boundaries, rural roads, and other features, and the overall accuracy of identification using high-resolution remote sensing data is improved by 14.1% compared with the overall accuracy of identification using MODIS data (Tian, 2019; Tian et al., 2019). Song et al. studied Landsat, Sentinel-2, Sentinel-1 and MODIS remote sensing data at different spatial resolutions for crop type mapping of soybean and corn in the Continental United States (Song et al., 2021). Another study evaluated the application efficiency and effectivity of rice mapping based on the GEE cloud platform in Southern Punjab, Pakistan, from coarse to fine resolution multispectral satellites (Sentinel-2 [10 m], Landsat-8 [30 m] and MODIS [250 m]) (Waleed et al., 2022). These studies proved that the higher the spatial resolution of optical remote sensing data is, the better the mapping accuracy of crop types is; moreover, Sentinel-2 data can effectively enable field-scale crop mapping, and the identification accuracy is affected by parcel size, planting density, and crop diversity. However, Landsat and Sentinel-2 data are usually studied in a small area due to their massive data volume (Belgiu and Csillik, 2018). After GEE was made available to the public in 2012, its powerful cloud computing capabilities have facilitated extensive, high spatial-resolution crop mapping based on Landsat and Sentinel series satellites. Gumma developed a spatial distribution map of agricultural land in South Asia on the basis of a machine learning algorithm by using Landsat time series data on GEE (Gumma et al., 2020). Nasrallah extracted the 10 m resolution winter wheat distribution in the Bekaa Valley of Lebanon by using Sentinel-2 time series data (Nasrallah et al., 2018). The use of remote sensing data with high spatial resolution helps alleviate the low identification accuracy caused by parcel fragmentation. In addition, high-spatial-resolution remote sensing images help describe farmland cropping patterns at the landscape level and achieve highly effective extraction of crop phenology information at the parcel scale (Pan et al., 2015; Qiu et al., 2020).

In North China, In North China, winter crops are usually sown from September to October, with seedlings emerging before the over-wintering period and harvested around June. Winter wheat, garlic, and rapeseed are the principal winter crops of the North China Plain, with winter wheat accounting for the majority of production (Dong et al., 2020). Winter wheat and garlic are the two primary crops grown throughout the winter in the Shandong region. However, existing studies have focused on winter wheat, and little importance has been given to remote sensing mapping and area estimation of garlic (Chai et al., 2019). Garlic’s economic value has increased recently, resulting in an increase in planting areas. Given that winter wheat and garlic have almost the same phenological period and vegetation characteristics, the phenomenon of “ different objects with the same spectra characteristics “ arises, and garlic is easily as winter wheat, which makes the identification of winter wheat erroneous.

In summary, to address the problems of early-season identification of winter wheat, such as weak vegetation signal, limited spatial resolution of remote sensing data, heavy reliance on training samples, and the “ different objects with the same spectra characteristics “ of winter wheat and garlic, this study has completed a refined early-season mapping of winter wheat in Shandong Province with 10-m resolution on the basis of the GEE cloud platform, by using Sentinel-2 time series data supplemented by Landsat-8 data. In the study, a phenology feature index was developed for weak vegetation signal enhancement of winter wheat, and early-season identification discriminative rules were designed in combination with other information for different periods of winter wheat. In addition, a unique spectral feature was developed based on the growth differences between winter wheat and garlic. The proposed method does not require training samples and can achieve refined early-season identification of winter wheat with automated premise. The winter wheat identification results can be advanced to 4-6 months before the harvesting period, with high identification accuracy.

## 2 Materials

### 2.1 Study area

**Figure 1:**
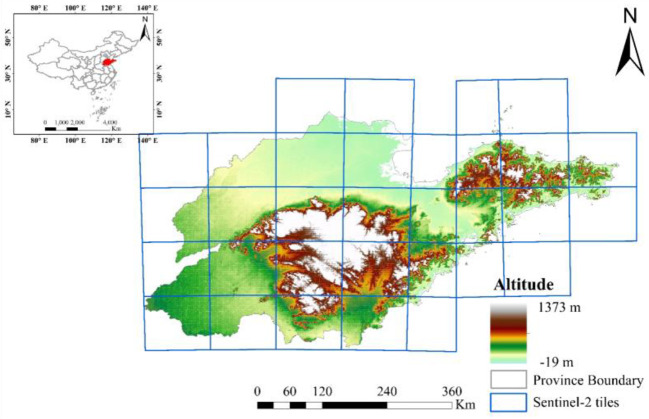
Location of the study area (It includes the location of Shandong Province in China, the coverage of Sentinel-2 relative orbits and the elevation data of Shandong Province).

Shandong Province is located on the eastern coast of China, and it is between 34°22′N-38°24′N and 114°47′E-122°42.3′E. It is situated in the lower reaches of the Yellow River and has a land area of about 155,800 km^2^, as shown in **Figure 1**. The climate of Shandong Province is warm-temperate monsoon, characterized by concentrated precipitation in summer, rain and heat in the same season, and coldness and dryness in winter. The average annual temperature is between 11°C and 14°C, and the temperature difference between the east and west is greater than that between the north and south. The annual precipitation is between 550 and 950 mm and decreases from southeast to northwest (Chen et al., 2012). The average annual amount of light is between 2,290 and 2,890 hours, and the sufficient heat conditions meet the needs of crops to mature twice a year (Xu et al., 2019). The central part of Shandong Province is mountainous, the southwest and northwest parts are low-lying and flat, and the eastern part is gently undulating. Plains account for 65.6% of the province’s area and are mainly located in the northwest and southwest of Shandong.

**Figure 2:**
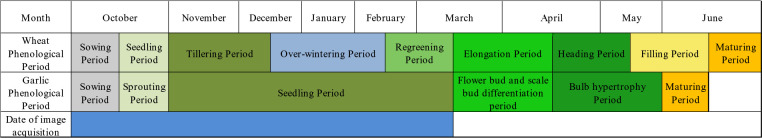
Winter wheat and garlic phenological calendar in Shandong Province.

The soil type in Shandong Province is mainly brown loam and brown soil. The fertile soil and good climatic conditions make Shandong Province the second major production center of winter wheat aside from Henan Province (Li et al., 2021). In 2021, 39,493,530,800 ha of winter wheat, which is the largest winter crop in Shandong Province, was planted in the said province Garlic, oilseed rape, vegetables, and greenhouse crops are also grown in the region, with garlic being the most widely planted. The phenological periods of winter wheat and garlic are basically the same; the sowing period is from September to October, and the harvesting period is in June. The specific phenological periods are shown in **Figure 2**.

### 2.2 Data and preprocessing

In this study, the processing and analysis of remote sensing images were carried out on the GEE, and the remote sensing data mainly included Sentinel-2A/B MSI data and Landsat-8 OLI data. This study also used SRTM elevation data, field sample data, and winter wheat planting area statistics The elevation data were directly derived from GEE (https://developers.google.com/earth-engine/datasets/catalog/USGS_SRTMGL1_003), and the statistical data were obtained from Shandong Provincial Bureau of Statistics (http://tjj.shandong.gov.cn/tjnj/nj2021/indexch.htm).

#### 2.2.1 Remote sensing image data

This study used two types of Sentinel-2 optical images are used in the study, which are surface reflectance data and top-of-atmosphere reflectance data. The surface reflectance data were employed to construct a time-series winter wheat phenology curve, and the top-of-atmosphere reflectance data were adopted to extract winter wheat phenology characteristics. Both types of data were pre-processed via radiometric calibration, geometric correction, and topographic correction, and the temporal resolution of the data was five days. Landsat-8 surface reflectance data were invoked on the GEE cloud platform and had already been pre-processed via radiometric calibration and geometric correction. The main purpose of these data was to compensate for the lack of effective observations of Sentinel-2 optical images due to poor weather, with a revisit period of 16 days. The spectral parameters of Landsat-8 and Sentinel-2 are shown in **Table 1**.

**Table 1:** Remote sensing image waveband parameters.

Bands Name	Landsat-8 wavelength range (μm)	Sentinel-2 band center wavelength (S2A/S2B)
Aerosols	B1(0.43~0.45)	B1(443.9nm/442.3nm)
Blue	B2(0.45~0.51)	B2(496.6nm/492.1nm)
Green	B3(0.53~0.59)	B3(560nm/559nm)
Red	B4(0.64~0.67)	B4(664.5nm/665nm)
NIR	B5(0.85~0.88)	B8(835.1nm/833nm)
SWIR 1	B6(1.57~1.65)	B11(1613.7nm/1610.4nm)
SWIR 2	B7(2.11~2.29)	B12(2202.4nm/2185.7nm)
Red Edge 2		B5(703.9nm/703.8nm)
Red Edge 2		B6(740.2nm/739.1nm)
Red Edge 3		B7(782.5nm/779.7nm)
Red Edge 4		B8A(864.8nm/864nm)

#### 2.2.2 Field sampling data source

The data on winter wheat sample points were obtained from the field survey of the national agricultural department, and the sampling period was between November and December 2021, with a total of 3,885 winter wheat sample points in Shandong Province. Samples of other surface types were automatically generated in GEE by using a sampling method. The detailed operation is to used ESA-2020 and AGLC-2015 as the base data, and calculate the concatenation of cropland types in the two datasets was calculated to define them as cropland areas. Then random points were generated in the non-cultivated area, and the land type and coordinate information of the random points were extracted In the end, a total of 2,500 random non-wheat sample points were generated. A total of about 6,385 sample points were obtained for the accuracy verification of the identification results.

#### 2.2.3 Detection of clouds and cloud shadows

In this study, cloud and cloud shadow removal was performed in accordance with the clouds presenting different features from other ground features in an image (Lewis and Brown, 2001). The details of the process are as follows: Clouds are bright and moist in the cirrus band, blue band, and all visible bands, so the cloud score of the image is calculated and the cloud probability is detected using data from four bands (aerosol, blue, green and red bands) together with two spectral indices (normalized difference humidity index and normalized difference snow index). Cloud shadows are then judged based on the solar geometry features and the position clouds, so clouds and cloud shadows can be detected and removed accurately at the same time. This method is more effective than the QA60 band for cloud and shadow removal (You and Dong, 2020).

## 3 Methods

### 3.1 Weak signal enhancement in early winter wheat

At the early stage of winter wheat growth, the winter wheat leaves are small and cannot cover the ground completely. The winter wheat vegetation signal is also mixed with background information, such as soil and snowmelt. The vegetation signal features on remote sensing images are not noticeable due to the background information, and the vegetation changes shown on time-series images are less sensitive because of the slow growth of winter wheat at the early growth stage. A new vegetation index called normalized differential phenology index (NDPI) was selected in this research to enhance the weak vegetation signal characteristics at the start of winter wheat growth (Wang et al., 2017). NDPI can suppress soil and snow cover compared with the traditional vegetation index and can reduce the influence of weak vegetation response at the early stage of winter wheat growth and winter snow. NDPI is calculated as shown in Equation 1:


(1) $$NDPI=\frac{\delta_{NIR}-(\alpha \times \delta_{RED}+(1-\alpha )\times \delta_{SWIR})}{\delta_{NIR}+(\alpha \times \delta_{RED}+(1-\alpha )\times \delta_{SWIR})}\text{,}$$

where *δ_NIR_* is the NIR band reflectance, *δ_RED_* is the red band reflectance, *δ_SWIR_* is the shortwave infrared reflectance, and *α* is set at 0.74 since it is the most potent value for reducing changes in the soil and snow backdrop (Wang et al., 2017).

**Figure 3:**
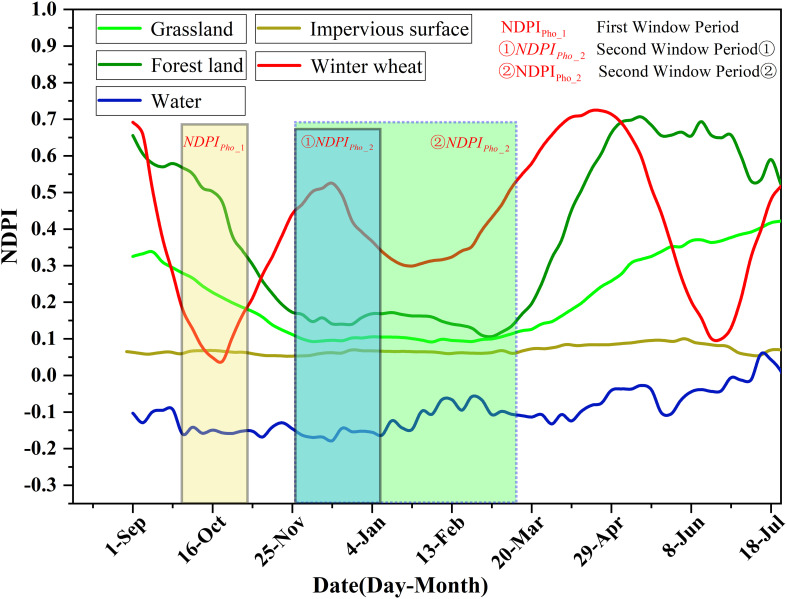
Time-series NDPI curves for winter wheat and other landforms.

Extraction of time series variation characteristics based on NDPI was performed using Sentinel-2 data. First, cloud and cloud shadow removal and five-day-interval time-series NDPI index median composition were conducted. Second, NDPI index reconstruction was performed using the Savitzky-Golay filtering algorithm. Lastly, NDPI feature images with a five-day interval at 10 m spatial resolution were acquired (Savitzky and Golay, 1964). In the study area for winter wheat and other major surface types (including woodlands, grasslands, water bodies, and impervious surfaces), 100 sample points were evenly selected for each surface type. Their mean values were calculated to generate the phenological curves and conduct a comparative analysis, as shown in **Figure 3**.

The analysis of **Figure 3** shows that, the first window period was in the winter wheat sowing period (light yellow background), where the NDPI value of winter wheat was the smallest, that is, about 0.1. The NDPI values of forest and grassland (i.e., about 0.3) were higher than that of winter wheat. The NDPI values of impervious surfaces was low, generally less than 0.1, and the NDPI value of water bodies was less than 0. The second window period ① (blue background, black solid line area)was the early over-wintering period and over-wintering period of winter wheat, in which the chlorophyll content of winter wheat increased, so and the NDPI value also increased. The NDPI of winter wheat was greater than 0.35. The NDPI values of water bodies and impermeable surfaces were mostly unchanged, whereas those of forest, grassland, and other withering vegetation typically decreased to below 0.2. The second window period ② (the region with a green background and a blue dotted box) had a start time that was similar to that of the second window period ① and an end time that was the regreening period of forest and grassland (before March 15). At this time, winter wheat had entered the regreening period for 2_3 weeks. At the end of the second window period ②, the NDPI value of winter wheat increased from a low value during the over-wintering period to be at par with the maximum value of NDPI during the early over-wintering period, with an NDPI value greater than 0.45. During the second window period ②, forest and grassland had just entered the regreening period; their NDPI values increased slightly, but most of the forest and grassland NDPI values were still in the low-value period. The NDPI value of impervious surfaces slightly increased because the vegetation in the residential land returned to green and formed a mixed image with the buildings. The NDPI value of water bodies slightly increased but was lower than 0.1, which could be well distinguished.

To further enhance the weak vegetation signal characteristics of winter wheat and highlight the time-series vegetation variation of winter wheat, this study proposed two phenological indices for early-season identification of winter wheat based on the phenological characteristics of early winter wheat growth; the two indices are winter wheat phenology differential Index (WPDI) and the normalized differential wheat phenology index (NDWPI). The formulas are as follows:


(2) $$WPDI=Max\left\{NDPI_{Pho\_2}\right\}-Min\left\{NDPI_{Pho\_1}\right\}\text{,}$$

(3) $$NDWPI=\frac{Max\left\{NDPI_{Pho\_2}\right\}-Min\left\{NDPI_{Pho\_1}\right\}}{Max\left\{NDPI_{Pho\_2}\right\}+Min\left\{NDPI_{Pho\_1}\right\}}\text{,}$$

where *NDPI_pho__*_1_ denotes the time series data of NDPI in the first time window of winter wheat, *NDPI_pho__*_2_ denotes the time series data of NDPI in the second time window of winter wheat.

*Min*{*NDPI_pho__*_1_} denotes the minimum value of the NDPI calculated in the first time window, and *Max*{*NDPI_pho__*_2_} denotes the maximum value of the NDPI calculated in the second time window. The settings of the time windows are shown in **Figure 3**.

In addition, the day of year (DOY) corresponding to the maximum value of NDPI in the early over-wintering period is set as an important feature for judging winter wheat.


(4) $$DOY_{\max}=\arg\max\left\{NDVI_{pho}|pho_{\_1}\leq pho\leq pho_{\_2}\right\}\text{,}$$

where *DOY*_max_ is the DOY corresponding to the maximum value of NDPI, *pho___*_1_ corresponds to the winter wheat sowing period, *pho___*_2_ corresponds to the winter wheat early over-wintering period, and argmax{*f*(*x*)} indicates the *x* corresponding to the maximum of *f*(*x*), that is, DOY.

The DOY corresponding to the maximum value of NDPI represents the date of the most vigorous growth of winter wheat. According to the winter wheat growth phenology calendar and the analysis of **Figure 3**, the peak growth date of winter wheat is generally after mid-November, and the peak growth period of woodland and grassland is before winter wheat sowing. Therefore, DOY can be used to help distinguish winter wheat from woodland and grassland.

In this study, NDPI was used to mitigate the mixed pixel effect and enhance the weak vegetation signal in the early growth period of winter wheat. Two feature indices, WPDI and NDPI, were also developed based on the phenological characteristics to increase the distinguishability of winter wheat from other ground features in the time dimension. However, because garlic and winter wheat have similar phenological characteristics, they cannot be effectively distinguished from each other by using the abovementioned method.

### 3.2 Spectral characterization of winter wheat and garlic

**Figure 4:**
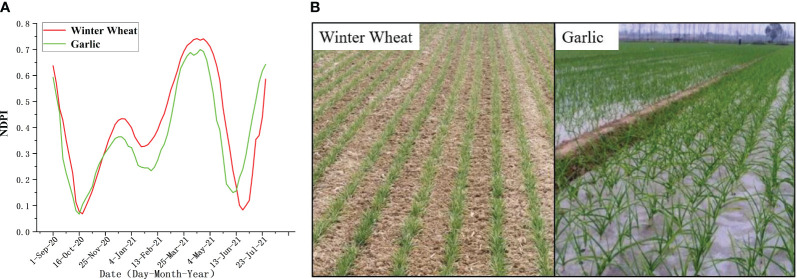
Differences between winter wheat and garlic [**(A)** displays the phenology curves for winter wheat and garlic, and **(B)** displays fieldwork photos of winter wheat and garlic at the early growth stage].

Winter wheat and garlic have similar phenological characteristics, and both belong to green vegetation, as shown in **Figure 4A**. The two time-series NDPI curves in this study were similar, although garlic had slightly lower NDPI values than winter wheat throughout the growth stage. However, some variations in NDPI values without obvious boundaries were observed. Additionally, the harvest period for garlic was a little bit earlier than the harvest period for winter wheat, but considering the length of the harvest period and the interval satellite observations, precisely differentiating between the two based on vegetation or phenological characteristics was challenging. Nevertheless, garlic and winter wheat have a few different planting practices, though. Winter wheat is more resistant to cold than garlic is, so when garlic is seeded, the mulch is often covered to prevent frost damage during the overwintering season, as shown in **Figure 4B**.

**Figure 5:**
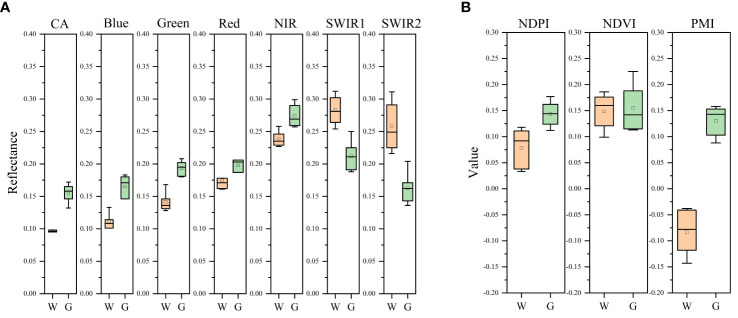
Spectral analysis of winter wheat and garlic W represents winter wheat, G represents garlic, **(A)** shows the spectral band information, and **(B)** shows the spectral index information].


In this study, a mulch characteristic index was created to differentiate garlic from winter wheat by using the specific qualities of plastic mulch utilized during the early stages of garlic growth. In this study, 100 sample points from the winter wheat-and garlic-growing regions were collected for spectrum analysis through a field survey and visual interpretation of high-resolution Google pictures. The data are displayed in **Figure 5A**. The plastic mulch used at the earliest stage of the garlic planting process is often colorless and transparent, and the ground surface it covers has higher brightness characteristics than bare soil. The primary chemical component of the plastic mulch is polyvinyl chloride, which has a higher transmittance in the 1500–2500 μm range than the visible near infrared band in terms of spectral characteristics. **Figure 5A** shows that although the reflectance values of plastic mulch decreased in the short-wave infrared band range, they increased in the visible to near-infrared band range in the garlic-growing region compared with the winter wheat-growing area. In the study, a plastic mulched index (PMI) was developed based on the special spectral characteristics of plastic mulch. PMI is expressed as

(5) $$PMI=\frac{\delta_{NIR}-\delta_{SWIR1}}{\delta_{NIR}+\delta_{SWIR1}}\text{,}$$

where *δ_NIR_* is the reflectance of the NIR band and *δ_SWIR_*_1_ is the reflectance of the shortwave infrared1 band. The reflectance of the NIR and shortwave infrared1 bands was chosen in this study because the PMI results are highly stable with a small range of data fluctuations while maintaining a large difference.

As illustrated in **Figure 5A**, discerning between winter wheat and garlic by using NDPI and NDVI vegetation indices is difficult. Given that PMI data fluctuate less and the feature is stable, using the PMI feature index suggested in the study allows for easy distinction between winter wheat and garlic as a contemporaneous crop. The PMI feature index is negative in areas where winter wheat is grown and positive in areas where garlic is grown.

### 3.3 Decision tree algorithm classification

The decision tree technique is a popular classifier for classifying remote sensing images. The fundamental idea behind this algorithm is to create a set of rules using expert knowledge, divide them into levels in accordance with the tree structure, and make logical decisions at each level in accordance with the rules until the classification is complete at the final leaf node. The decision tree method has a clear structure, good interpretability, and quick and easy operation (Friedl and Brodley, 1997).

**Figure 6:**
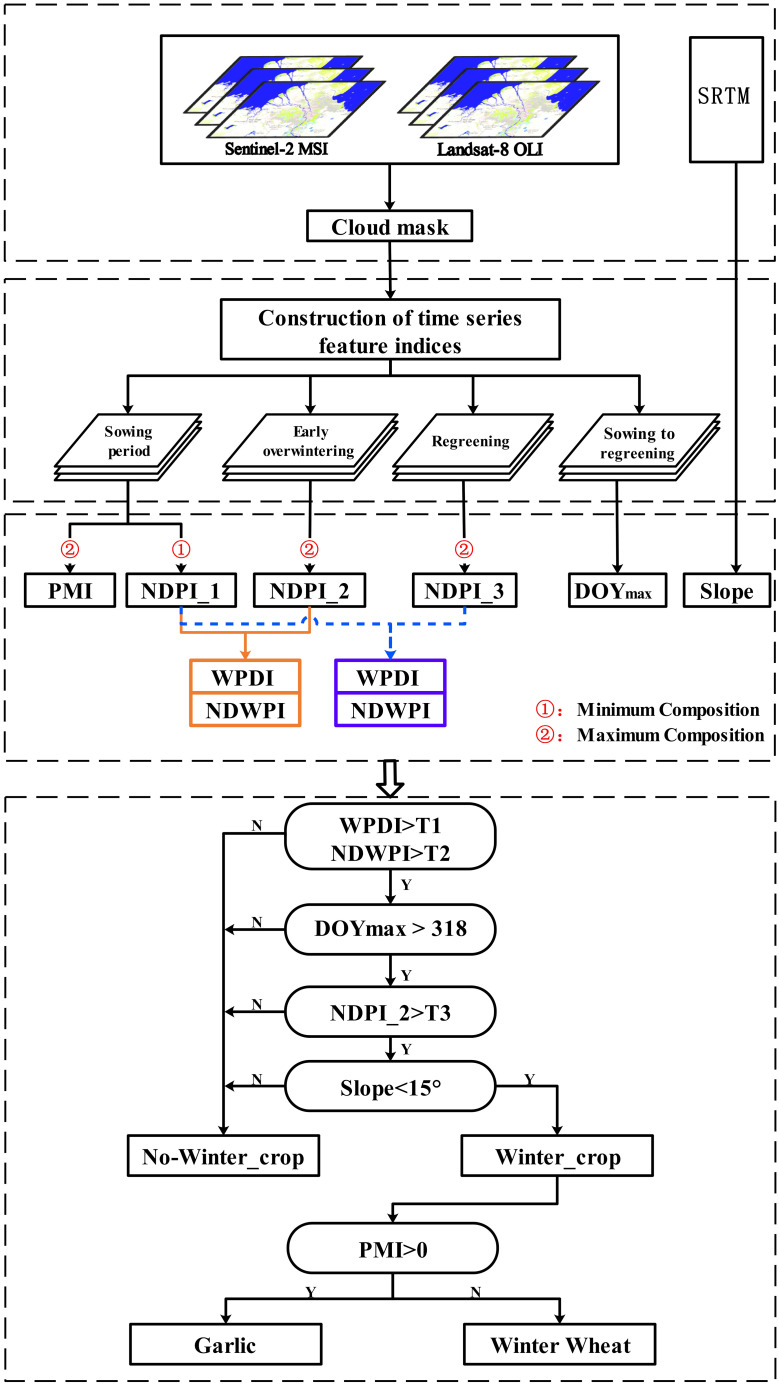
Flow chart of early refinement mapping of winter wheat with the decision tree algorithm.

On the basis of the findings presented in Sections 4.1 and 4.2, a tree was constructed for refined early-season mapping of winter wheat in this study, and the classification rules of the decision tree are illustrated in **Figure 6**.

First, removal of clouds and cloud shadows was performed on the GEE platform for Sentinel-2 and Landsat-8 data. Second, a time-series vegetation index dataset was constructed. Owing to the influence of cloud removal, remote sensing images in some areas or times have no-data values. In this study, the median composition algorithm was used to generate spatio-temporal continuous data at five-day intervals. The three phenological periods’ time windows were selected based on the results of the analysis in Section 4.1 and **Figure 3**, and the defining images of each period were filtered in accordance with the time windows.

Image composition based on the time window of the winter wheat phenological period can enhance the image features of early winter wheat growth and reduce data redundancy. We completed the minimum composite of time-series NDPI in the sowing period, the maximum composite of time-series NDPI in the early over-wintering period, and the maximum composite of time-series NDPI in regreening period. From to the winter wheat phenology calendar and phenology curve in Shandong Province, we found that NDPI reached the maximum value in the tillering period, and we set the threshold value of DOY corresponding to NDPI to 318. DOY greater than the threshold value of 318 refers to a winter crop. Winter wheat is often planted in plain areas with mild slopes, and less land is cultivated in central and eastern mountainous areas (Valero et al., 2021). In addition, 15° is used as the slope threshold, and winter wheat is grown in areas where the slope is less than 15°. Other characteristics including water bodies, forests, grasslands, and impervious surfaces may be distinguished based on the five abovementioned rules to extract the spatial distribution of winter crops, but garlic, a contemporaneous crop of winter wheat, cannot be extracted.

On the basis of the spatial distribution map of winter crops, garlic was distinguished from winter wheat by using the PMI index developed in this study. With the analysis conclusion in Section 3.2, the PMI maximum composite of the sowing period was calculated, and the area with PMI greater than 0 was established as the garlic planting area with mulching. The other areas were winter wheat planting areas.

Automated early-season refined mapping of winter wheat was achieved in all regions of Shandong Province without a large number of training samples and by using only *a priori* knowledge of winter wheat phenology.

### 3.4 Accuracy assessment

Accuracy verification is necessary to evaluate the recognition performance and is mainly divided into type accuracy and quantitative accuracy. Type accuracy is usually assessed using the confusion matrix. Quantitative accuracy describes how closely a particular type area matches the actual area. In this study, the overall area accuracy was used to evaluate the quantitative accuracy of recognition, and the area consistency index was adopted to evaluate the consistency between the extracted area and the actual area; the higher the area consistency was, the higher the extraction accuracy was.

The confusion matrix is generally calculated based on the acquired samples and classification results, and the specific evaluation metrics include overall accuracy (OA), user accuracy (UA), production accuracy (PA), kappa coefficient, and the derived F1 score (Lewis and Brown, 2001). OA and kappa are used to evaluate the overall classification results, and the F1 score is a combined metric consisting of PA and UA; it provides a comprehensive evaluation of each type of classification accuracy.


(6) $$OA=\frac{\sum_{i=1}^{n}A_{ii}}{N}\times 100\%\text{,}$$

(7) $$Kappa=\frac{N\sum_{i=1}^{n}A_{ii}-\sum_{i=1}^{n}\left(A_{i+}\times A_{+i}\right)}{N^{2}-\sum_{i=1}^{n}\left(A_{i+}\times A_{+i}\right)}\text{,}$$

(8) $$PA=\frac{A_{ii}}{A_{+i}}\times 100\%\text{,}$$

(9) $$UA=\frac{A_{ii}}{A_{i+}}\times 100\%\text{,}$$

(10) $$\text{F}1\_Score=2\times \frac{\text{PA}*\text{UA}}{\text{PA}+\text{UA}}\text{,}$$

where n represents the number of classes, and it is also the total number of rows or columns of the matrix; *A_ii_* represents the number of image elements on the first row and column; and N represents the total number of real samples. *A_i+_* represents the sum of the pixels on row *i*, and *A_i+_* represents the sum of the pixels on column *i*.

Total area accuracy (TA) refers to the closeness of the extracted area to the true area. The true area uses the yearbook results published by local and municipal statistical offices, as follows:


(11) $$TA=1-\frac{\left|X-Y\right|}{X}\times 100\%\text{,}$$

where X is the statistical area in the yearbook and Y is the area of the extraction results.

Area consistency accuracy is achieved through a regression analysis of the crop area published in the yearbook of the municipal statistical office with the results of identification, and calculation of the coefficient of determination (R^2^) (Phalke et al., 2020). Large values indicate a high correlation of the dependent variable, which also indicates good area consistency between the extracted area and the statistical data. A comparison is then made between the linear fit line and the 1:1 line, which is used to reflect the direction of deviation. R^2^ is shown as


(12) $$R^{2}=1-\frac{\Sigma {\left(y_{i}-\hat{y_{i}}\right)}^{2}}{\Sigma {\left(y_{i}-\bar{y_{i}}\right)}^{2}}\text{,}$$

where y*_i_* is the identification area of the *i* municipal unit, *ŷ* is the statistical area, and *ȳ* is the average of the area.

## 4 Results

### 4.1 Winter wheat and garlic early-season mapping

**Figure 7:**
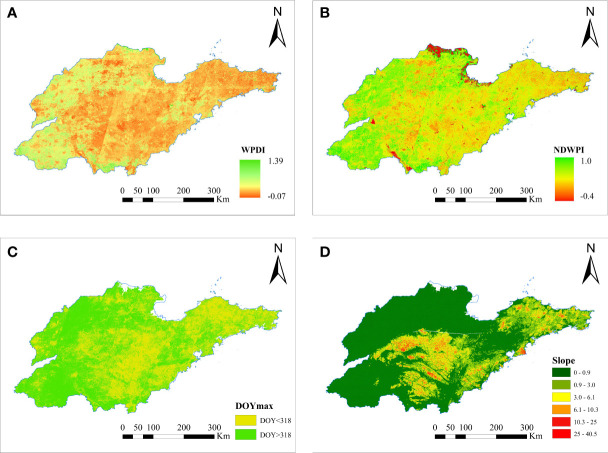
Example of decision rule feature image. [**(A–D)** respectively show the images of four feature indices: WPDI, NDWPI, DOY max, and slope].

The judgment indices for constructing the decision tree are shown in **Figure 7**. For the two characteristic indices, WPDI and NDPI, the results obtained from the sowing and early over-wintering periods were selected as examples. Their large values indicated that the vegetation change was great and that the increasing trend of NDPI was obvious. Although the NDPI of winter wheat increased a little relative to that of garlic, the distinction between the two was not obvious because both winter wheat and garlic were green in the early over-wintering period. The comparison showed that the trend of DOY max had a strong correlation with the trend of WPDI and NDPI, and the macroscopic change trend was consistent.

**Figure 8:**
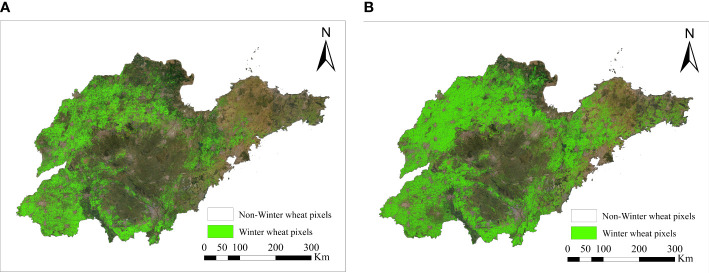
Spatial distribution of winter wheat in Shandong Province.

On the basis of the decision tree algorithm, **Figure 8A** shows the identification results of the early over-wintering period with the date of January 15, and **Figure 8B** shows the identification results of the regreening period with the date of March 15. The identified winter wheat area in the regreening period was slightly larger than the identified area in the early over-wintering period, and the increased area was mainly in the eastern part of Shandong Province. A comprehensive analysis revealed that the winter wheat planting areas in Shandong Province were mainly distributed in the western, northern, and southern regions, in which four cities, namely, Heze, Dezhou, Liaocheng, and Jining, had large and concentrated planting areas. This distribution was mainly due to the wide distribution of mountains and hills in the central and northeastern regions of Shandong, which are unsuitable for the cultivation of winter wheat. Moreover, the soil texture in the eastern coastal region was relatively poor, and the planting suitability of winter wheat i was low; hence, the planting area of winter wheat was small and scattered.

**Figure 9:**
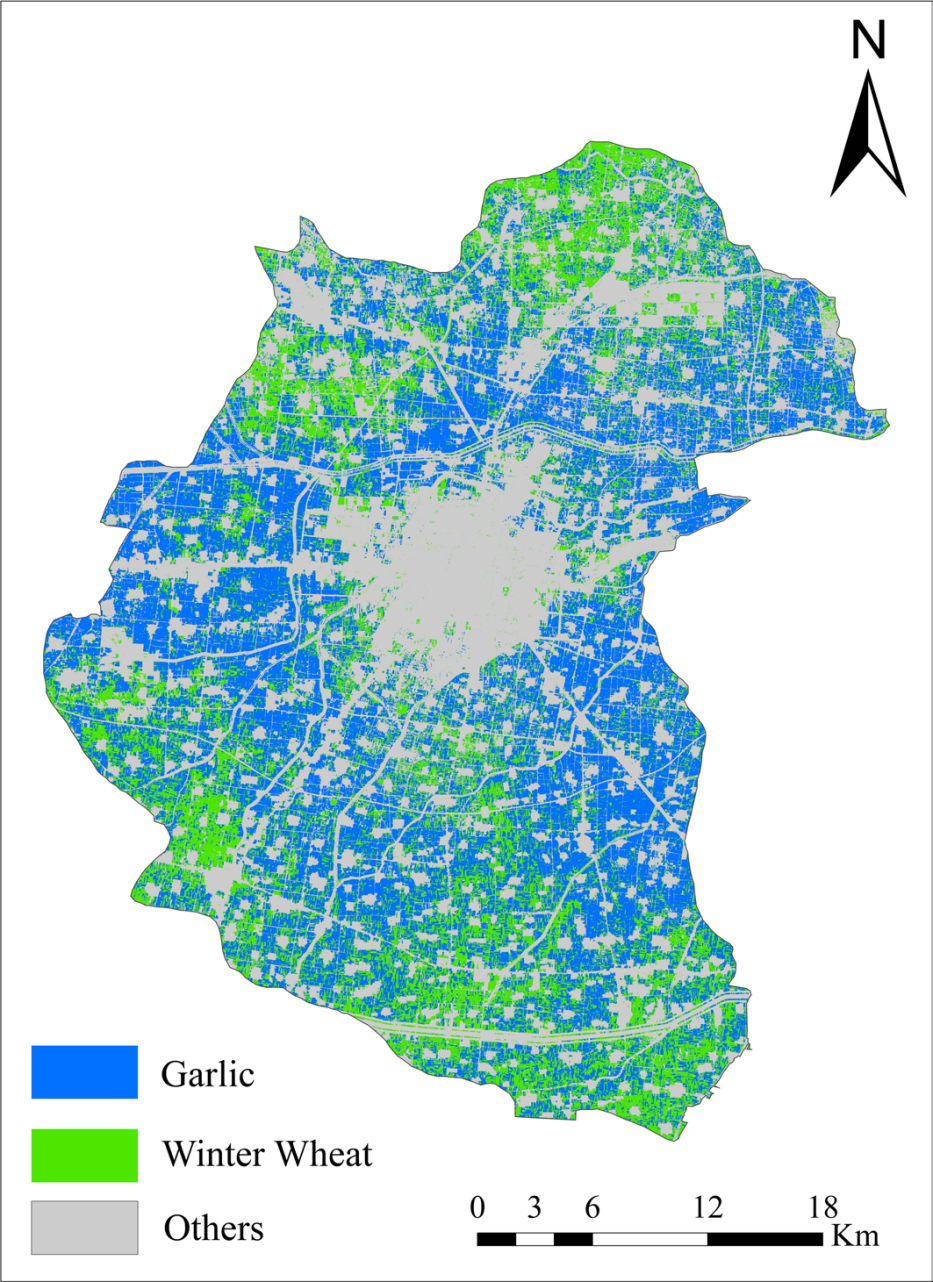
Early-season identification results of winter wheat and garlic in Jinxiang County.

This study completed the early spatial distribution of winter wheat in Shandong Province, and also acquired the spatial distribution map of early mapping of garlic. Garlic cultivation in Shandong Province was mainly distributed in Jinxiang County of Jining City and its surrounding areas, among which Jinxiang had the widest garlic cultivation area and the most concentrated distribution. Meanwhile, garlic cultivation in the rest of the areas was relatively fragmented. In this study, we adopted Jinxiang County of Jining as an example to show the identification results of garlic, as indicated in **Figure 9**. Garlic is the main plantation in Jinxiang County, and the distribution of garlic is relatively concentrated. The main plantation zones are the central, eastern, and western parts. The distribution of winter wheat is fragmented, and the planting area is mostly in long and narrow patches. The distribution of winter wheat in the north and south of Jinxiang County is relatively concentrated, which is basically consistent with the background survey of crop cultivation.

### 4.2 Accuracy verification

Early-season identification mapping of winter wheat was conducted in two phenological periods, namely, early over-wintering and regreening. We used three methods for accuracy evaluation: calculation of the confusion matrix, statistical area validation of municipal administrative units, and zoomed maps of local areas.

#### 4.2.1 Validation on sample points

**Figure 10:**
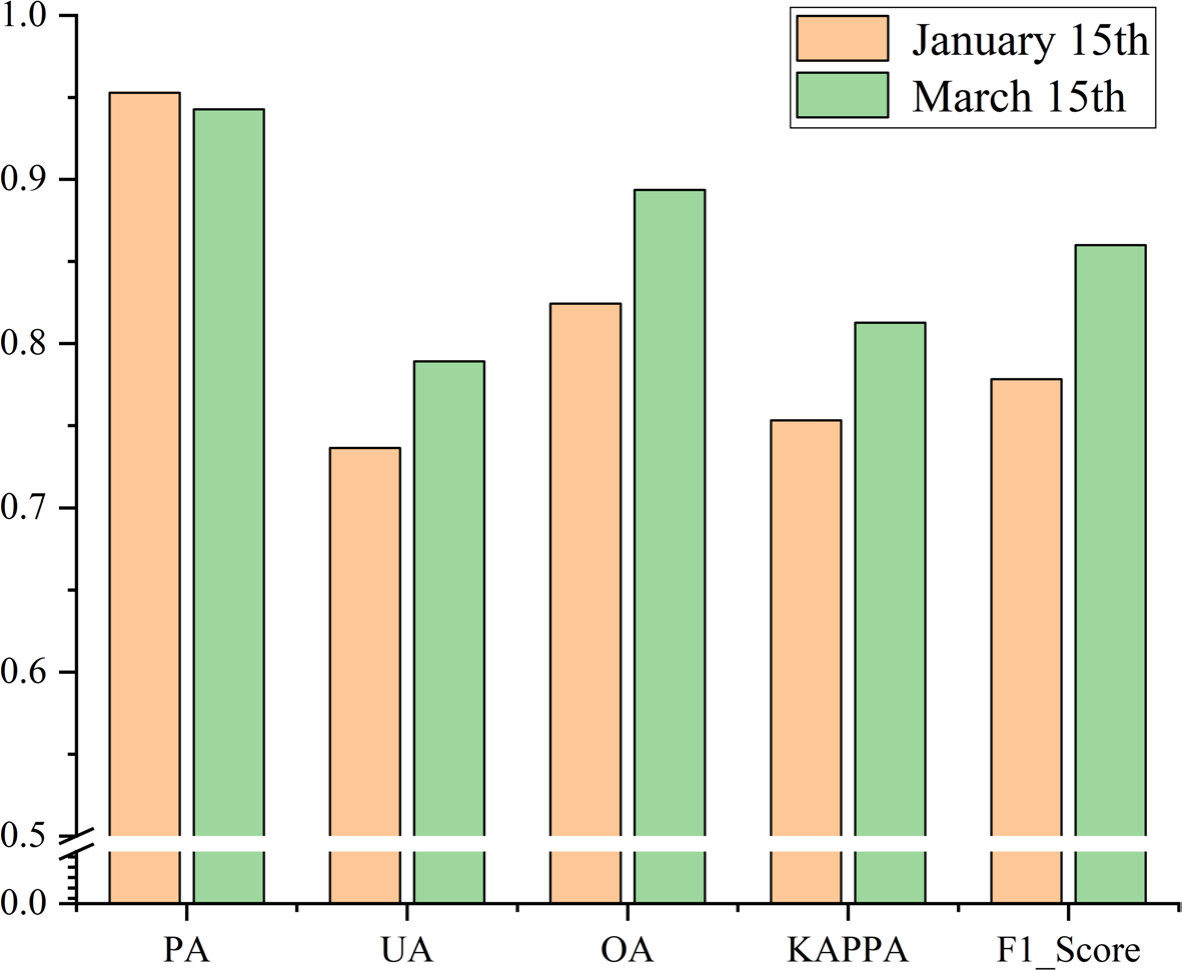
**(A, B)** Evaluation results of the confusion matrix for early-season identification of winter wheat.

On the basis of the data of 3,885 winter wheat sample points and 2,500 non-winter wheat sample points, the accuracy of the early-season identification results of winter wheat was evaluated by calculating the confusion matrix for the early over-wintering and regreening period. The results are shown in **Figure 10**. The analysis revealed that the accuracy of the early-season identification results of winter wheat in the regreening period was higher than that in the early over-wintering period, with an overall accuracy of 82.64%, F1 score of 0.84, and kappa coefficient of 0.78. The accuracy of the identification results in the regreening period was improved to some extent, with an overall accuracy of 88.76%, F1 score of 0.89, and kappa coefficient of 0.84. The reason for the higher accuracy of the identification results in the regreening period is that the growth of winter wheat in the regreening period is better and more uniform than that in the early over-wintering period, and the vegetation signal of wheat is more prominent. In conclusion, the early-season identification results of winter wheat in the study have high accuracy.

#### 4.2.2 Verification of official statistics

**Figure 11:**
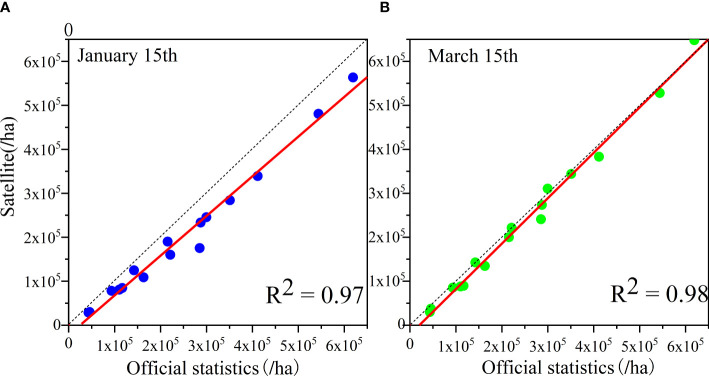
**(A, B)** Verification of consistency between the identification results and official statistics.

To further verify the identification accuracy, this study calculated the planted area in accordance with the early-season identification results of winter wheat. The official statistics of winter wheat planted area in Shandong Province in 2021 was 3,949,353 ha. Meanwhile, the identified area in the early over-wintering period was 3,206,666.8 ha and the identified area in the regreening period was 3,990,686.7 ha; the overall area accuracy of the two periods was 81.2% and 98.9%, respectively. The identification results in the early over-wintering period were underestimated, and the identification results in the regreening period were overestimated. The reasons for the underestimated results in the early over-wintering period include the inconsistent growth of winter wheat in this period and the late sowing and un-sowing, which lead to missed identifications. The overestimated results in the regreening period may be due to the situation of winter wheat being harvested for green storage before the harvest stage, in addition to misclassification. Another main reason is that the area of winter wheat has been reduced by natural meteorological disasters or pests’ diseases. Furthermore, to validate the consistency of the identification results, this study verified the area of winter wheat identified in this work based on official statistics in 16 municipalities in Shandong Province. The results are shown in **Figure 11**. The analysis revealed that the early-season identification results of winter wheat in both periods had good consistency, and the R^2^ values were both above 0.95. The identified area in the over-wintering period was slightly lower than the statistical area.

In this study, we could not obtain garlic field sample points, so the confusion matrix was not calculated for accuracy evaluation. This study adopted Jinxiang County, a large garlic planting county, as an example to verify the statistical area. A total of 35,933.5 ha of garlic was planted in Jinxiang County in 2021, and the early-season identification area of garlic in the study was 31,666.8 ha, with an overall area accuracy of 88.13%. After excluding the garlic planting area, the overall accuracy of the winter wheat area identification was improved from 65.2% to 96.8%, which increased the identification accuracy.

#### 4.2.3 Classification result subset analysis

**Figure 12:**
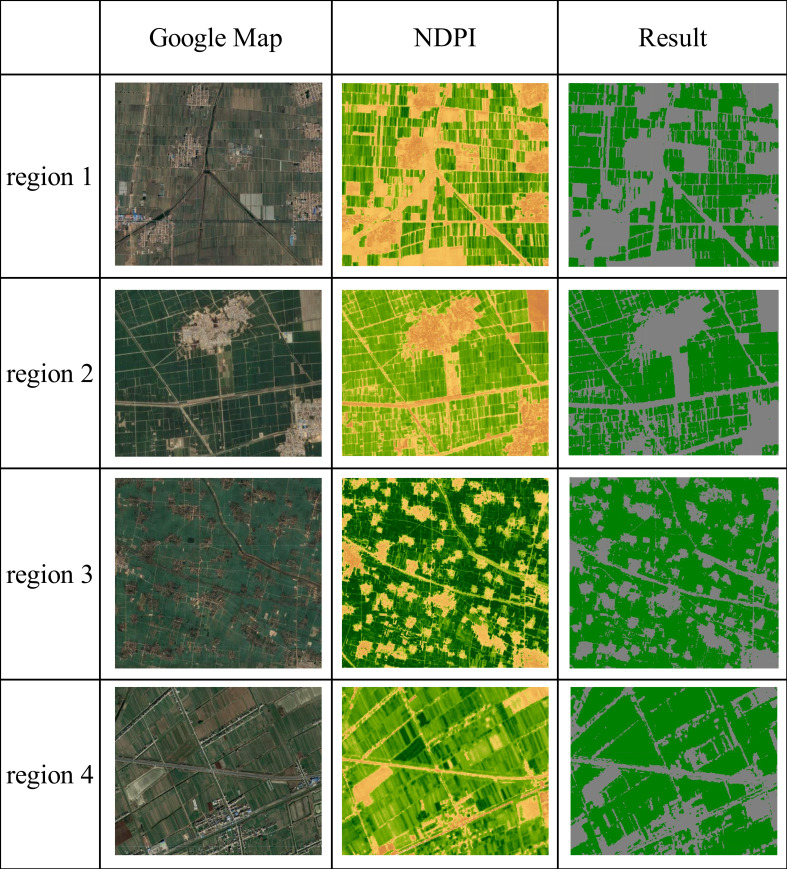
Validation of winter wheat identification results.

As shown in **Figure 12**, the early-season identification results of winter wheat in the northern, eastern, southern and central-eastern parts of Shandong Province were randomly selected with one sample each, and the Google image was obtained from Google Earth with a resolution of 1 m. The NDPI image was the result obtained from using the Sentinel-2 image in the April elongation period with a resolution of 10 m. The remote sensing features of winter wheat in this period were relatively obvious. The third column shows the early-season identification results. The results indicate that the recognition of winter wheat distribution based on the Sentinel-2 image with 10 m resolution was good, and roads, settlements, bare land, water bodies, and wide farmland boundaries could be distinguished. The identified farmland details were relatively rich, which cannot be achieved by lower-resolution remote sensing data.

**Figure 13:**
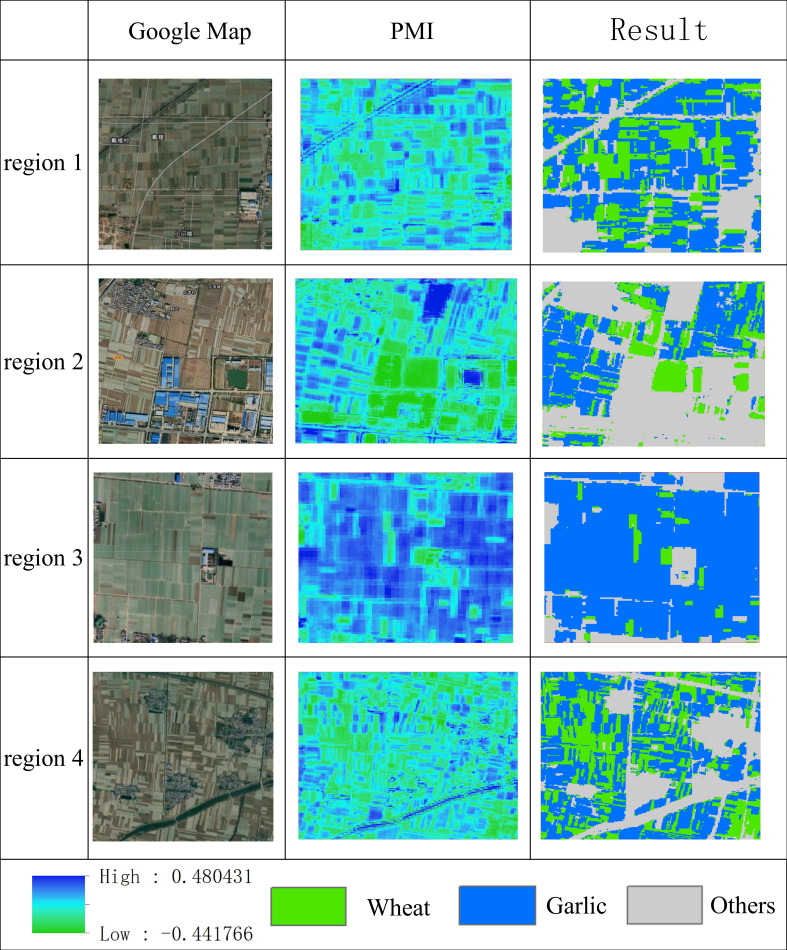
Validation of winter wheat and garlic identification results.

To further evaluate the effectiveness of this study on differentiating winter wheat and garlic, four validation samples were selected in Jinxiang County for an accuracy assessment, as shown in **Figure 13**. The first column is the Google image, the second column is the calculated PMI index, and the third column is the identification result.

The results of the four validation regions showed that the method in the study could identify garlic and winter wheat well. Region 1 had an interspersed distribution of garlic and winter wheat, and the PMI index could be used to distinguish them well. Region 2 contained many other objects, such as buildings, water bodies, bare land and woodland. The developed method could also accurately identify garlic and wheat without interference from the other objects. Region 3 was mainly a large garlic planting area, and the spatial continuity of the recognition results was good, almost without the interference of noise points, and some main roads between fields could be recognized. Region 4 mainly had an interlaced distribution of winter wheat and garlic, and many narrow parcels had high fragmentation. From the analysis, we found that the recognition effect for narrow fields was not ideal, but the overall recognition accuracy basically met the requirements.

## 5 Discussion

### 5.1 Comparison and analysis of vegetation indices

At present, the main approach to extract phenological information based on remote sensing technology is to construct time-series curves from vegetation indices, and vegetation indices are often used as the main discriminative feature for crop identification. Common vegetation indices include RVI, NDVI, and EVI (Huete et al., 2002; Ji and Peters, 2003). The main goal of was to achieve an early-season identification of winter wheat. Winter wheat grows slowly at the early stage of growth, and the leaf area is small and cannot cover the ground completely, resulting in a weak vegetation signal; meanwhile, the traditional vegetation index is affected by soil and snow cover (Dong et al., 2020). To verify the advantage of NDPI over other vegetation indices in enhancing the weak signal characteristics of vegetation in winter wheat at the early growth stage, this study used NDVI and EVI for a comparative analysis. NDVI and EVI are calculated as follows:


(13) $$NDVI=\frac{\delta_{NIR}-\delta_{RED}}{\delta_{NIR}+\delta_{RED}}\text{,}$$

(14) $$EVI=2.5\times \frac{\delta_{NIR}-\delta_{RED}}{\delta_{NIR}+6\times \delta_{RED}-7.5\times \delta_{BLUE}+1}\text{,}$$

where *δ_NIR_* is the NIR band reflectance, *δ_RED_* is the red band reflectance, and *δ_BLUE_* is the blue band reflectance.

**Figure 14:**
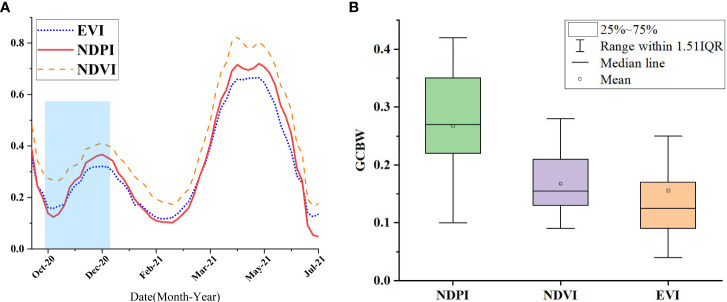
Comparison of vegetation indices for winter wheat. [**(A)** displays the Winter wheat phenology curve, **(B)** displays the Vegetation index GCBW statistical results].


In the study area, 200 winter wheat sample points were selected. We constructed the phenological curves of NDPI, NDVI and EVI. The results are shown in **Figure 14A** that NDPI had a more sensitive response to vegetation changes in early winter wheat compared with the other vegetation indices. To quantitatively evaluate the change in the three vegetation indices, the quantitative evaluation index of Greenness Change Before Winter (GCBW) was defined in this study with the following equation 15.The results are shown in **Figure 14B**. The analysis showed that NDPI had the largest amount of variation, with a mean value of about 0.25. NDVI and EVI had a mean value of about 0.15, and the mean value of NDVI was slightly higher than that of EVI. The results of the quantitative analysis showed that NDPI index could more effectively reflect the changes in vegetation at the early stage of wheat growth compared with the other indices. This finding fully supports the selection of NDPI in this study. NDPI can effectively enhance the weak signal characteristics of winter wheat vegetation at the early stage of growth, and it has greater advantages in early mapping of winter wheat compared with NDVI and EVI.

(15) $$GCBW=VI_{\max}-VI_{\min}\text{,}$$

where *VI_max_* is the maximum value of the vegetation indices before the over-wintering period, and *VI_min_* is the minimum value of the vegetation indices before the over-wintering period.

### 5.2 Transferability analysis

**Figure 15:**
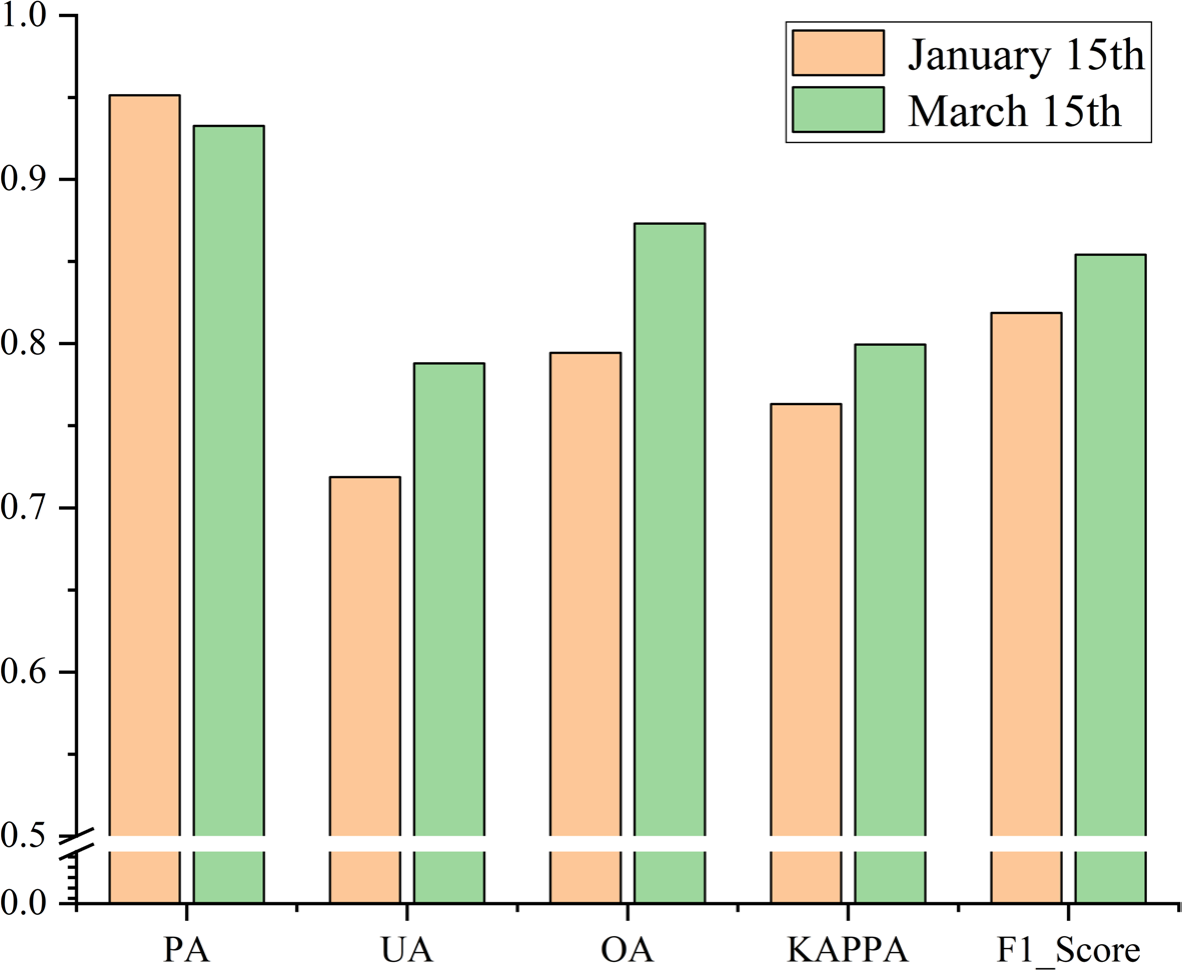
Evaluation results of the confusion matrix for early-season identification of winter wheat in 2020.

To more comprehensively evaluate the migration ability of the model in the study, we generalized the model in temporal and spatial dimensions to verify its effectiveness in early-season winter wheat refinement identification in other years and other regions. We extended the model to the early-season refinement identification of winter wheat in Shandong Province in 2020, and because the crop types in Shandong Province hardly change from year to year, we used the winter wheat samples in 2021 to verify the accuracy. The results are shown in **Figure 15**. The results of the confusion matrix calculation were as follows. In the early over-wintering period the overall accuracy was 79.43%, the F1 score was 0.82, and the kappa coefficient was 0.76. The accuracy of identification in the regreening period was improved compared with that in the early over-wintering period. The overall accuracy was 87.31%, F1 score is 0.85, and Kappa coefficient is 0.80. The accuracy of early-season winter wheat identification in 2020 was slightly reduced compared with that in 2021. Specifically, the overall accuracy was reduced by about 2% on the average, the F1 score and kappa coefficient were reduced by 0.03 on the average, and the identification effect was basically unchanged. The slight decrease in accuracy may be due to the differences in winter wheat sowing dates between years and differences in winter wheat growth due to different temperatures and precipitation. Nevertheless, the model still exhibited a strong transfer ability between years.

**Figure 16:**
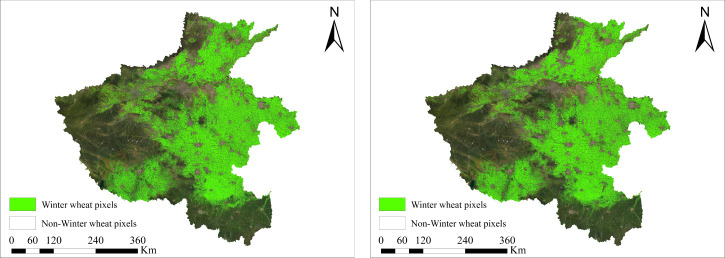
Spatial distribution of winter wheat in Henan Province.

To verify the ability to transfer geospatially, the model was applied to Henan Province, the largest winter wheat growing area in China, for the early-season refinement identification of winter wheat. Henan Province, located in the west of Shandong Province, has the largest winter wheat cultivation area and production in China. The climate is basically similar to that of Shandong Province, which has a warm temperate monsoon climate, and the province is mostly a plain area, which is highly favorable for the cultivation of winter wheat in large areas. And there are nearly have 800,004,000 ha of garlic planting area in Qixian and Zhongmou counties in Henan province. The planting structure of winter crops in Henan Province is basically the same as that of Shandong Province. The early-season mapping of winter wheat in the early over-wintering (January 15) and regreening (March 15) periods was completed using the developed method, and the identification results are shown in **Figure 16**. The analysis showed that the identification results of the two periods were basically the same, but the identification area of the regreening period was slightly higher than that of the early over-wintering period. Notably, winter wheat in Henan Province is mainly planted on the eastern plains, and the area planted in western hilly areas is relatively small.

**Figure 17:**
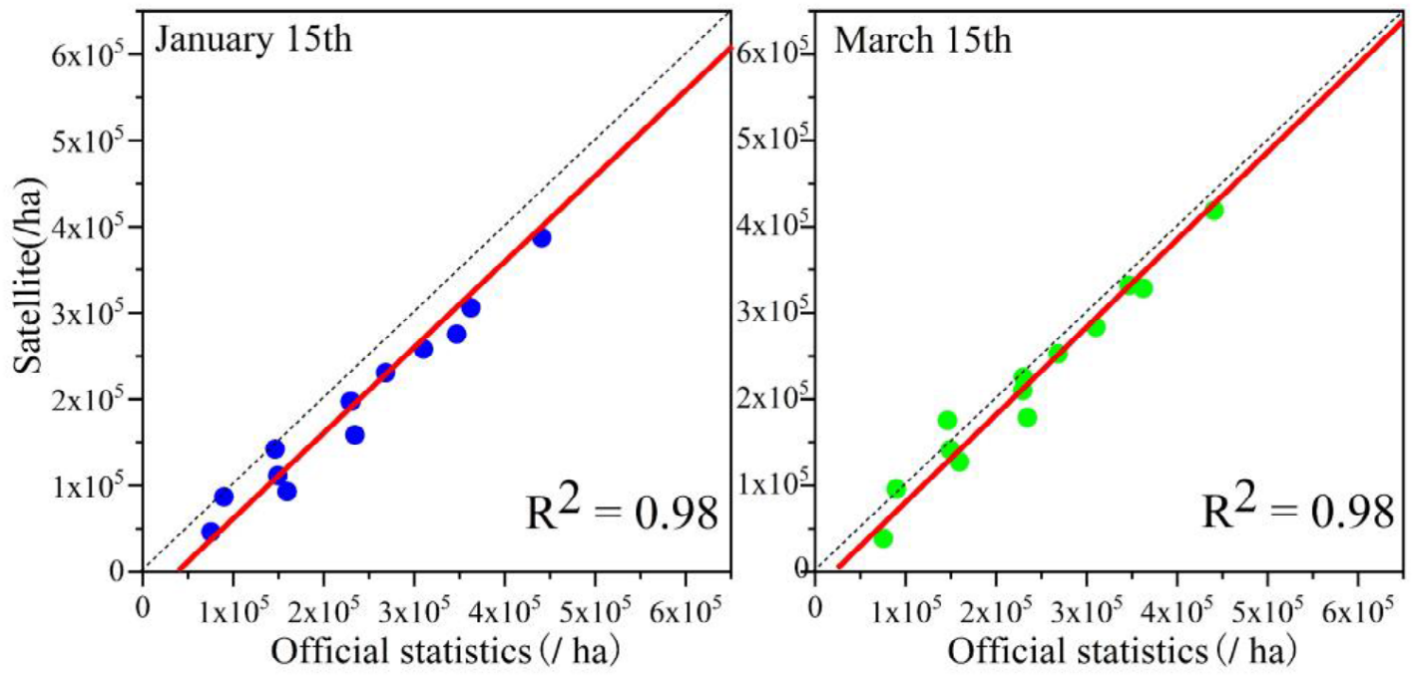
Verification of the consistency between winter wheat identification results and official statistics in Henan Province.

Given that a sufficient amount of field sample point data in Henan Province could not be obtained, this study used only the official statistics released by Henan Province for accuracy verification. A consistency test between the early-season identification results of winter wheat and statistical data was conducted for 17 prefectural cities in Henan Province, and the results are shown in **Figure 17**. The early-season identification results of winter wheat in Henan Province in both periods showed high identification accuracy and were in good agreement with the official statistics; the R^2^ of both reached 0.98. According to the official statistics, 6,102,030 ha were planted with winter wheat in Henan Province in 2021, and the identified winter wheat area in the study was 5,465,360 ha in the early over-wintering period and 5,901,362.8 ha in the regreening period, with an overall area accuracy of 89.6% and 96.3%, respectively. The recognition accuracy in the early over-wintering period in Henan Province was even better than that of Shandong Province possibly due to the wider plain area in Henan Province, better natural conditions for winter wheat cultivation, and higher degree of mechanized cultivation compared with Shandong Province. Thus, the developed method of this study has strong spatial generalization ability and can be applied to other regions for high-precision early-season winter wheat refinement recognition under automated methods.

### 5.3 Impact factors of classification results

**Figure 18:**
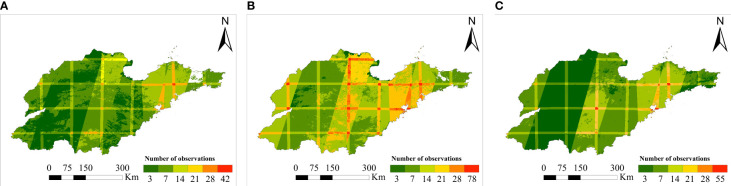
Availability of Sentinel-2 images. [**(A)** corresponds to the temporal window of the sowing period, **(B)** corresponds to the temporal window of the early over-wintering period, and **(C)** corresponds to the temporal window of the regreening period].

The decision tree model constructed in the study based on *a priori* knowledge such as winter wheat phenology information, as a fully automated classification method, has a great advantage in early-season refinement recognition of winter wheat. It can complete the mapping of winter wheat spatial distribution 4-6 months before the winter wheat harvest period, and the method has a strong generalization ability. In this study, we showed through an experimental analysis that the present method can be extended to temporal and spatial dimensions, and can complete the refined mapping of winter wheat in historical years and other regions with high recognition accuracy. Compared with supervised classification methods (e.g., random forest and support vector machine), the developed method does not require the manual labeling of a large number of training samples (Dong et al., 2020; Zhang et al., 2021). Although the developed method is not limited by samples, the classification results are affected by the number of effective observations of images, spatial inconsistency of winter wheat phenology and other factors (Sun et al., 2012). Valid observation data on complete and intensive time series could not be obtained due to the influence of the revisit cycle and cloudy and rainy weather, so this study could not complete winter wheat mapping in the whole area of Shandong Province by using Sentinel-2 data. We plotted the number of effective observations in Shandong Province based on the time window of the phenological period for early-season mapping of winter wheat, as shown in **Figure 18**. The larger the number of effective observations, the more abundant the vegetation information on the early growth of winter wheat is and the better the mapping results are. However, as shown in **Figure 18**, the number of effective observations in some areas was fewer than three, reflecting little information on winter wheat, and this situation is not conducive to the identification of winter wheat. Therefore, supplementation with Landsat-8 data is necessary to increase the number of effective observations, that is, to increase the number of acquired cloud-free images.

In this study, the rules for building the decision tree were mainly based on phenological information at the early stage of winter wheat growth, and the precision of the phenological information determined the accuracy of the mapping. In Shandong Province, the eastern part is adjacent to the sea, the western part is a plain, and the central part is hilly and mountainous; thus, the phenological information is relatively complex. To verify the differences in winter wheat phenology at early growth stages, this study extensively collected winter wheat phenology information from all counties in the province. The results showed that the DOYmax corresponding to the maxima NDPI of winter wheat in the early over-wintering period ranged from 335 to 352, and the spatial distribution exhibited the patterns of being later in southern Shandong than in northern Shandong and being later in the inland than in the coast. The DOYmax corresponding to the regreening periods was between 45 and 65, and the spatial distribution of NDPI in the greening period was earlier in southern Shandong than in northern Shandong and earlier in the inland than in the coastal areas. The maximum difference in the phenology period in the different regions was about 20 days, and obvious spatial differences were observed, which made the effective extraction of winter wheat features difficult. To reduce the errors caused by inconsistent phenology, this study adopted the method of time window synthesis to extract the most obvious phenological features for enhancing the vegetation characteristics of the early growth stage of winter wheat. The decision tree algorithm built based on multiple rules in this study is affected by other factors, such as crop conditions, sowing dates, and natural conditions, but it has a good generalization ability. Delayed sowing and poor weather conditions, such as temperature and precipitation, can lead to poor crop growth when the NDPI increase in winter wheat is reduced. In addition, when cultivated land has poor soil fertility or insufficient sunlight, the growth of winter wheat can be reduced. In particular, winter wheat is grown at low latitudes under satisfactory natural conditions such as sufficient light, temperature, and precipitation, and winter wheat phenology may skip the over-wintering period and grow non-stop, during which the threshold needs to be adjusted.

### 5.4 Outlook

The proposed method has some advancements and advantages in early-season identification of winter wheat, but it also has some limitations that can be further improved in future research. First, only NDPI and its derived phenological feature indices were used in the study. This limitation can be further investigated in the future via multi-feature fusion or by developing other effective weak vegetation signal feature enhancement indices. Second, the decision tree algorithm used in the study requires manual setting of thresholds, which are based on the results of the phenology analysis and may change under varying external conditions (e.g., temperature, precipitation and anthropogenic factors). Hence, a dynamic adaptive threshold method could be developed to further improve the automation and applicability of the model. Third the phenology of the study area presents some inconsistencies. When early-season identification of winter wheat in a large area is carried out, partitioning based on the phenological differences should be considered to ensure the consistency in all zones, which is conducive to achieving high-precision, automated, early-season identification in large areas. Fourth, with the in-depth application of deep learning technology in remote sensing, deep learning methods can now automatically extract the features of images and distinguish the importance of features autonomously. Therefore, we can conduct the early-season identification of winter wheat by using deep learning methods (Xu et al., 2021). At the same time, we can perform research on the early-season identification algorithm of winter wheat on a large scale by using domestic satellite images.

## 6 Conclusions

Using the GEE cloud platform and the Sentinel-2 and Landsat-8 remote sensing data provided by it, this paper investigates the theory and algorithm of early-season refinement mapping of winter wheat by analyzing the characteristics and phenological features of winter wheat of early growth stage. As an example, early-season refinement mapping of winter wheat with 10m resolution was completed in Shandong Province, and the earliest identification time was 4_6 months before the harvesting period. The main conclusions are as follows:

(1) In response to the weak remote sensing response of the vegetation signal in the early period of winter wheat growth, an enhanced index of the weak vegetation signal characteristics of winter wheat was developed. Two winter wheat phenological feature indices, namely, WPDI and NDPI, were developed to enhance winter wheat information, and applied to the early-season identification of winter wheat. The indices achieved good identification effects.
(2)The problem that winter wheat and garlic are difficult to be distinguished from each other due to the “different objects with the same spectra characteristics” phenomenon was solved. By analyzing the planting habits and quantitative spectral differences between winter wheat and garlic, this study developed a PMI index that can effectively achieve early-season identification of winter wheat and garlic. Ideal early mapping results on garlic were obtained for Shandong Province (especially in Jinxiang County).
(3). An automated algorithm for early refinement of winter wheat mapping was constructed based on winter wheat phenological information. The overall accuracy of the model was 82.64% and 88.76% in the early over-wintering and regreening periods, respectively. The identification results showed good agreement with the official municipal statistics, with the R^2^ of the two being 0.96 and 0.98, respectively. We also performed temporal and geospatial transfer learning by using our algorithm and proved its effectiveness and strong generalization capability for large-scale early-season mapping of winter wheat.

## Data availability statement

The original contributions presented in the study are included in the article/**Supplementary Material**. Further inquiries can be directed to the corresponding author.

## Author contributions

XYL: conceptualization, investigation, methodology, writing - original draft, writing - review & editing. XHL, LG and JZ: resources, supervision, writing - review & editing. DQ and KW: resources, data curation. ZL: resources, formal analysis, writing - review & editing, supervision. All authors contributed to the article and approved the submitted version.
