# Supplementary material for: Early-season and refined mapping of winter wheat based on phenology algorithms - a case of Shandong, China
Source: Front Plant Sci. 2023 Jul 24;14:1016890. doi: 10.3389/fpls.2023.1016890 (PMC10405738; doi:10.3389/fpls.2023.1016890)
Supplement: Supplementary file 1 [file Table_1.docx]

Supplementary Material

# Supplementary Figures and Tables

## Decision tree algorithm classification

Where T1 is 0.2, T2 is 0.42, T3 is 0.32, the threshold value of DOY max is 318, the slope is 15°, and the threshold value of PMI is 0.0.


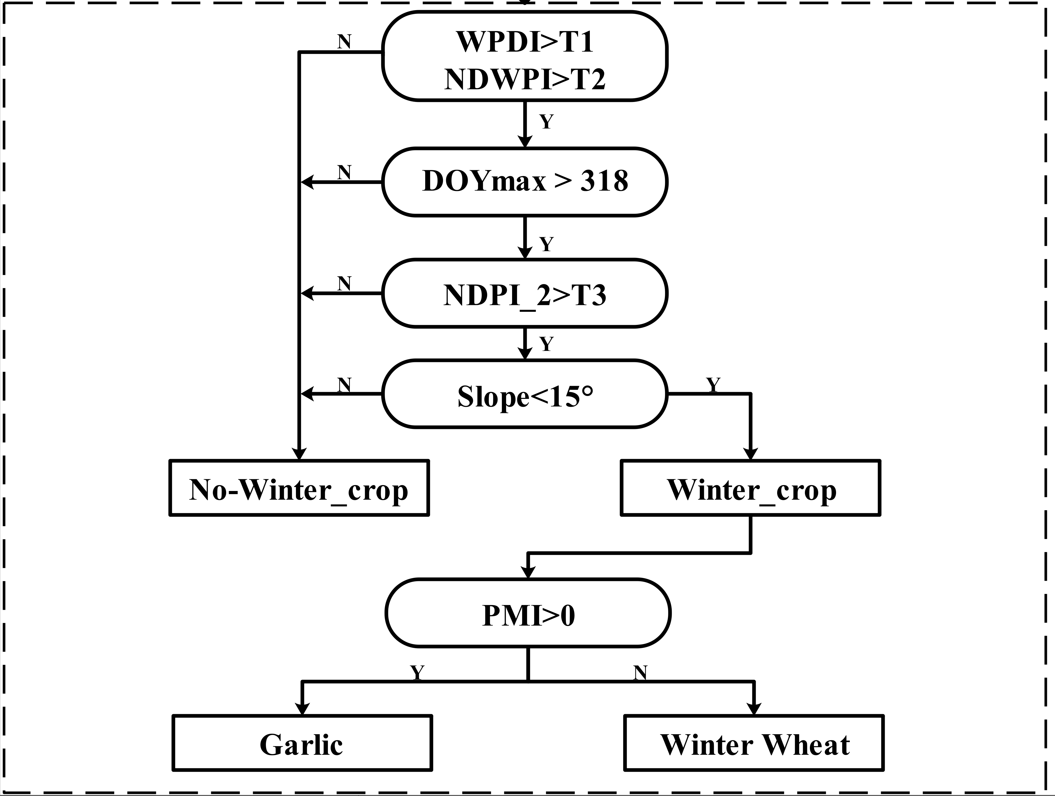


Decision tree model (corresponds to Figure 6 in the original paper)

**A demo code in GEE is as follows：**

1. **var** wheat = WPDI.gt(0.2).and(NDWPI.gt(0.42))
2. .and(DOY.gte(318))
3. .and(NDPI_2.gt(0.32))
4. .and(slope.lt(15))
5. .and(PMI.lt(0.0))
7. **var** garlic = WPDI.gt(0.2).and(NDWPI.gt(0.42))
8. .and(DOY.gte(318))
9. .and(NDPI_2.gt(0.32))
10. .and(slope.lt(15))
11. .and(PMI.gte(0.0))
